# 

**Published:** 1894-08-11

**Authors:** 


### CONTENTS.

**Canon Ainger's True Physician** - 385

**Around the Hospitals** - 386

**The Ethylates, or Caustic Alcohols.** By Sir Benjamin Ward Richardson, M.D., F.R.S. - 387

**Some Aspects of Medical Science** - 388

**Annotations.**—The Babies of Battersea—Influenza, A Theory of Cause and Cure—Medical Ambitions - 389

**Notes and News** - 390

**The British Medical Association** - 391

**Medical Progress and Hospital Clinics:**

Innocent Tumours of the Rectum - 395

Periscope of Dermatology - 396

**The Institutional Workshop:**

Practical Departments - 399

Construction Notes - 399

**Scraps and Gleanings** - 400

**Continental Notes from a Holiday Correspondent.** Baden-Baden - 401

### EXTRA SUPPLEMENT.—THE NURSING MIRROR.

**News from the Nursing World.**—Nurses' "At Homes"—A Lesson to All—Distribution of Diets—The Management of Measles—"Trained Nurse" for Shropshire—A Sensible Entertainment—Wisbech—Barry Dock—Llanrhos—Qualified at Boyle—Brisbane "Infants" as Nurses—Guides to Knowledge - cxc

**On General Nursing.**—XXIV. Typhoid Fever (*continued*) cxciii

**Structure and Care of the Teeth.**—I. Development - cxciv

**A Case for Assistance** - cxciv

**Character Sketches from a Lunatic Asylum** - cxcv

**Where to Go** - cxcv

**Cholera Precautions** - cxcvi

**The Bedford Workhouse** - cxcvi

**Appointments** - cxcvi

**Women's Work.** Women and Physical Education - cxcvii

**Hints on Disinfecting Rooms** - cxcvii

**Notes and Queries** - cxcviii

**For Reading to the Sick** - cxcviii

**The Muses' Looking Glass.** Round the London Hospital - cxcix

**Everybody's Opinion** - cxcix

**The Book World for Women and Nurses** - cc

